# Epigenetic Regulation of Pulmonary Arterial Hypertension-Induced Vascular and Right Ventricular Remodeling: New Opportunities?

**DOI:** 10.3390/ijms21238901

**Published:** 2020-11-24

**Authors:** Jordy M. M. Kocken, Paula A. da Costa Martins

**Affiliations:** 1 Department of Molecular Genetics, Faculty of Sciences and Engineering, Maastricht University, 6229 ER Maastricht, The Netherlands; j.kocken@maastrichtuniversity.nl; 2 CARIM School for Cardiovascular Diseases, Faculty of Health, Medicine and Life Sciences, Maastricht University, 6229 ER Maastricht, The Netherlands; 3 Unidade de Investigação Cardiovascular, Departamento de Cirurgia e Fisiologia, Faculdade de Medicina, Universidade do Porto, 4200-319 Porto, Portugal

**Keywords:** right ventricle remodeling, hypertrophy, pulmonary arterial hypertension, non-coding RNAs, epigenetics

## Abstract

Pulmonary artery hypertension (PAH) is a rare chronic disease with high impact on patients’ quality of life and currently no available cure. PAH is characterized by constant remodeling of the pulmonary artery by increased proliferation and migration of pulmonary arterial smooth muscle cells (PASMCs), fibroblasts (FBs) and endothelial cells (ECs). This remodeling eventually leads to increased pressure in the right ventricle (RV) and subsequent right ventricle hypertrophy (RVH) which, when left untreated, progresses into right ventricle failure (RVF). PAH can not only originate from heritable mutations, but also develop as a consequence of congenital heart disease, exposure to drugs or toxins, HIV, connective tissue disease or be idiopathic. While much attention was drawn into investigating and developing therapies related to the most well understood signaling pathways in PAH, in the last decade, a shift towards understanding the epigenetic mechanisms driving the disease occurred. In this review, we reflect on the different epigenetic regulatory factors that are associated with the pathology of RV remodeling, and on their relevance towards a better understanding of the disease and subsequently, the development of new and more efficient therapeutic strategies.

## 1. Pulmonary Hypertension 

### 1.1. Clinical Presentation

Pulmonary hypertension (PH) is a human pathophysiological condition defined by a mean pulmonary arterial pressure (mPAP) ≥25 mmHg at rest. PH can further be classified in five distinct groups, based on clinical presentation, overlapping hemodynamics and/or treatment strategies: pulmonary arterial hypertension (PAH), PH due to left heart disease, PH due to hypoxia or lung disease, PH due to chronic thromboembolism and PH due to unclear mechanisms [1]. This progressive disease is characterized by the remodeling of the pulmonary artery leading to vascular obstruction which, in turn, leads to increased blood pressure in order to preserve the blood flow [2,3]. PAH is further defined by a pulmonary capillary wedge pressure (PCWP) ≤15 mmHg and pulmonary vascular resistance (PVR) ≥3 Wood Units combined with mPAP ≥25 mmHg [1,4]. 

The disease can originate from idiopathic or heritable etiologies but can also be induced by certain drugs and/or toxins, or be associated with other diseases such as HIV, connective tissue, or congenital heart disease [1]. Currently, the prevalence of PAH in the western world is 2.7 per 100,000 individuals and is, therefore, considered as a rare disease, even though prevalence has been increasing in the last fifteen years [5,6]. Despite being rare, the disease highly impacts on the quality of life of the patients and subsequently on the healthcare system [7,8]. Patients with PAH display a plethora of symptoms varying from dyspnea, fatigue, swollen ankles and cyanosis [9], nevertheless diagnosis is challenging and involves multiple disciplines due to the many differences in etiologies. Patients with a history of hereditary PAH (hPAH) are tested for known genetic mutations in genes such as probable cation-transporting ATPase 13A3 (*ATP13A3*), bone morphogenetic protein receptor type II (*BMPR2*), Serine/threonine-protein kinase receptor R3 (*ACVRL1*), endoglin (*ENG*), mothers against decapentaplegic homolog 1, 4 and 9 (*SMAD1 SMAD4* and *SMAD9*), caveolin (*CAV1*), T-box transcription factor 4 (*TBX4)*, Eukaryotic translation initiation factor 2-alpha kinase 4 (*EIF2AK4*), Growth differentiation factor 2 (*GDF2*), aquaporin 1 (*AQP1*), SRY-box 17 (*SOX17*) and potassium two pore domain channel subfamily K member 3 (*KCNK3*), while simultaneously undergoing physical examination, exercise testing, biomarker analysis and right heart catherization [1,10,11,12]. 

**Figure 1:**
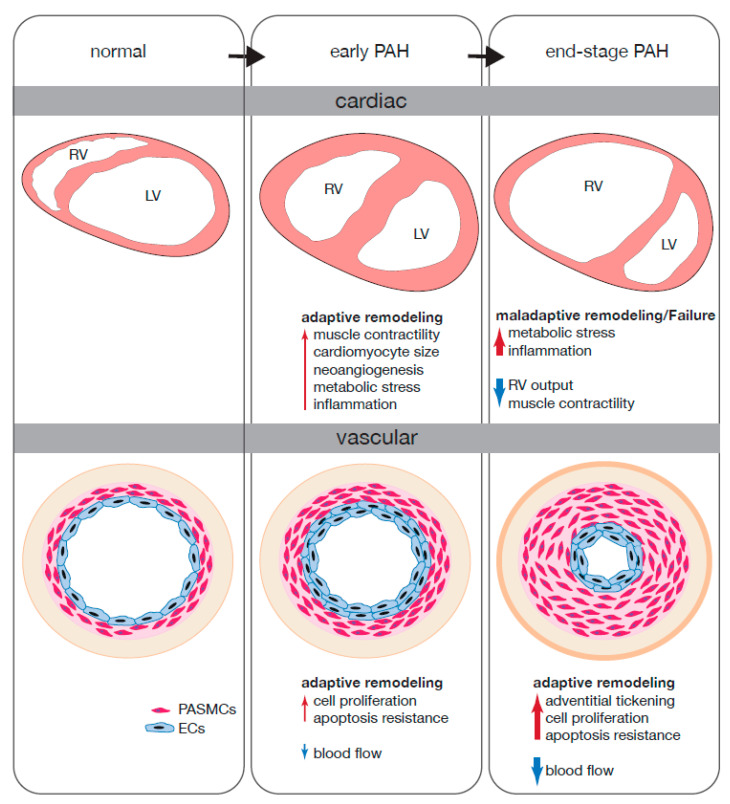
PAH induced pulmonary artery and subsequent RV remodeling and the ncRNAs involved in the process. During PAH, the pulmonary artery undergoes extensive remodeling due to increased proliferation and apoptosis resistance. The number of ECs and PASCMs increases which narrows the artery, decreasing the blood flow and increasing the pressure. Subsequently, the RV undergoes adaptive remodeling to overcome the increased PA pressure; during this adaptive remodeling, contractility, CM size, neoangiogenesis, metabolic stress and inflammation increases. During prolong PAH, the pulmonary artery continues to remodel with continuous proliferation, apoptosis resistance, blood pressure, and adventitial thickening while the blood flow decreases even further. Meanwhile, the RV can no longer sustain its output to overcome the pressure overload leading to a decoupling and maladaptive remodeling. This is marked by an increase in metabolic stress and further inflammation while contractility and RV output are decreased. The red arrows depict an increase of the mentioned change in homeostasis while a blue arrow signifies a decrease. Arrow thickness shows the severity of the change.

During PAH, the pulmonary artery undergoes chronic remodeling induced by constant proliferation of pulmonary endothelial cells (EC) and increased resistance to apoptosis which leads to an occlusion of the artery and subsequent pulmonary hypertension as depicted in Figure 1 [13,14,15]. 

The BMPR2 protein is known to be downregulated not only in the pulmonary vasculature of hPAH patients that harbor a mutation in the same gene, but also in patients that are suffering from a different etiology of the disease [16]. Although *BMPR2* mutations are not always prevalent in patients, the upstream targets may still be affected by certain cellular or molecular processes such as microRNA (miRNA) regulation [17] or viral HIV infection [18,19], all leading to lower BMPR2 protein levels and subsequent development of a pathophysiological response [20,21]. 

Whereas survival rates of patients are much dependent on the etiology of PAH and symptom severity, on average, a five-year survival rate between 40–60% is observed [22,23]. Roughly 33% of PAH patients succumb from right heart failure (RHF), a chronic end-stage cardiac disease originating from the inability of the right ventricle (RV) to compensate for the PAH [22,24].

### 1.2. Vascular Remodeling

Whereas PAH is characterized by progressive pulmonary vasculature remodeling, disease severity very much depends on its etiology, with patients harboring a genetic mutation being the most affected [25]. During the development of PAH, a plethora of pathways can be dysregulated. While here we focus on the three major signaling axis that are disrupted, namely the endothelial nitric oxide (NO), endothelin-1 (ET-1), and prostacyclin (PGI_2_) pathways [26,27,28], the contribution of other important pathways involving BMPR2/transforming growth factor beta (TGFβ) or RhoA/Rho-kinase (RhoA/ROCK) signaling have been extensively described in other reviews [29,30]. 

NO is produced in ECs by cleavage of the terminal amino group of the L-arginine amino acid by endothelial nitric oxide synthase (eNOS) and helps regulate the vasculature homeostasis [31]. Once generated, NO is transported outside the ECs where it will enter the bloodstream and influence neighboring smooth muscle cell function (SMCs) by regulating the formation of cyclic 3′,5′ guanosine monophosphate (cGMP) [32]. cGMP binds to either cGMP-dependent protein kinases, cGMP-binding phosphodiesterase or cGMP-regulated ion channels, leading to relaxation of the SMCs and lowering of blood pressure [33]. During PAH, NO has been suspected to be reduced in patients compared to healthy controls but while this holds true in some studies [26], other however, described increased eNOS levels in the pulmonary artery of hypertensive patients [34]. This discrepancy is also reflected in patients after treatment with NO inhalation, as younger patients of PAH benefit from NO administration while others do not respond to the treatment at all [35]. More recently developed therapies focus on the cGMP signaling pathway, either by inhibiting phosphodiesterase type 5 (PDE5), an enzyme that breaks down cGMP, or increasing the bioavailability of soluble guanylate cyclase (sGC), a source of cGMP [36]. However, and once again, not all patients benefit from the cGMP stimulators and while the reason why has not yet been elucidated, it may be attributed to different protocols and dosages used during the different clinical studies [37,38].

Another signaling pathway disrupted in PAH is the PGI_2_ signaling pathway. PGI_2_ originates mostly from ECs and has a vasodilating effect by activating specific cell surface receptors, mainly prostaglandin (IP) receptors, which are part of the G-coupled Protein Cell Receptors (GPCRs) family [39]. These receptors are coupled to activate adenylate cyclase which, in turn, converts adenosine triphosphate (ATP) into cyclic adenosine monophosphate (cAMP), a potent signaling molecule involved in multiple cellular processes including proliferation of pulmonary SMCs [40]. cAMP works through the activation of two downstream molecules, the exchange protein activated by cAMP (Epac) and Protein Kinase A (PKA) which work in synergy to inhibit extracellular signal-regulated kinases 1/2 (ERK1/2) and c-Jun N-terminal kinase (JNK) phosphorylation leading to inhibition of pulmonary SMC proliferation [41]. Epac was shown to be decreased in pulmonary artery smooth muscle cells (PASMCs) of PAH patients when compared to healthy controls and also in monocrotaline (MCT) rat models [42]. Targeted therapies for PGI_2_ have been available since 2009 and PAH patients administered with a continuous intravenous drip (24 h/day) of PGI_2_ have a higher improvement of clinical symptoms and survival rate, compared to patients receiving inhalation or oral administration of the drugs [43]. 

ET-1 is a 21 amino acid-long peptide, mainly released by the endothelium, with a potent vasoconstricting effect, opposed to NO and PGI_2_ which are vasodilating [44]. After being released by ECs, ET-1 is found to be circulating in the bloodstream in relatively low levels and while its levels can be measured to give insight in the general release of the peptide they do not account for the amount of peptide that is actually bound to cellular receptors [45]. ET-1 binds to two specific receptors, ET_A_ and ET_B_, on vascular SMCs and myocytes, with ET_B_ also being found on ECs [46]. Binding of ET-1 to its receptors in SMCs leads to activation of phospholipase C (PLC) and an increase in diacylglycerol and inositol triphosphate, followed by augmented intracellular calcium and subsequent incremented cellular contraction [47]. Increases of diacylglycerol and calcium in VSCMs also activates protein kinase C (PKC), suspected of activating a cascade of protein kinases and enhancing the cellular proliferative capacity [48]. Furthermore, activation of endothelial ET_B_ results in NO release and increased vasorelaxation in the pulmonary artery, but the direct influence of ET-1 in the process is considered to be minimal [49]. Current pharmacological treatment options available are ET_A_ and ET_B_ receptor antagonists, such as bosentan, macitentan or ambrisentan, while sitaxsentan has been withdrawn due to increased hepatic toxicity in patients [50]. Bosentan and macitentan act on both ET_A_ and ET_B_, despite a preference for blocking ET_A_, improving patient outcome parameters such as 6-min walking distance, dyspnea, and reducing clinical worsening [51,52,53]. Ambrisentan is a specific ET_A_ antagonist and is able to improve patient clinical outcome and quality of life while showing fewer side effects compared to bosentan or macitentan [54]. 

Unfortunately, ET-1 also affects pulmonary fibroblasts by inducing proliferation and increasing matrix deposition and stiffening in the pulmonary artery, further increasing the arterial pressure [55]. During PAH, not only the pulmonary circulation is affected by the hypertension, but recent studies suggest that vasculature dysfunction of the systemic circulation also develops. A recent study demonstrated that patients suffering from PAH, and more specifically idiopathic PAH (iPAH) or PAH patients with systemic sclerosis, display an increased risk of developing coronary artery disease (CAD), leading to lower survival expectations [56].

### 1.3. Right Ventricular Remodeling

During PAH, the RV undergoes prolonged pressure overload. Initially, and as an adaptive response to maintain the pulmonary circulation, the RV endures hypertrophic remodeling, characterized by an increase in cardiomyocyte (CM) size, increased intrinsic contractility and capillary angiogenesis as a way to provide sufficient oxygen and nutrients to the enlarged myocardium [57]. If pressure overload remains, as in PAH, the adaptive response gradually shifts to maladaptive remodeling towards RV failure [58] as depicted in Figure 1. The molecular and cellular responses that drive RV hypertrophy are distinct of what has been described for left ventricular (LV) hypertrophy [59]. As an example, during PAH, whereas chronic hypoxia induces activation of Hypoxia-inducible factor 1-alpha (HIF-1α) and leads to increased angiogenesis in the RV, pressure overload of the LV induces capillary rarefaction with decreased capillary networks surrounding the CMs [57,60]. Furthermore, while the LV stiffens and displays contractile dysfunction, the RV undergoes hypercontractile remodeling in order to preserve the pulmonary flow [61,62]. 

Hypercontractility of the RV leads to a shift in mitochondrial metabolism, from free fatty acid (FFA) oxidation to anaerobic glycolysis, to overcome the decreased supply of oxygen and to keep up with the increased energy demand of the CMs [63]. However, as glycolysis is normally used by a cell to overcome peak energy demands, switching to continuous glycolysis leads to chronic energy starvation in the myocardium [64]. During glycolysis, glucose enters the CM through the glucose transporter 1 (GLUT1) and glucose transporter 4 (GLUT4) glucose channels, to be processed further into pyruvate. Further processing leads to the production of ATP, through the Krebs cycle, or to lactate formation [65]. Previous studies using MCT or pulmonary artery banding (PAB), two models of PH in rats, showed that upon RV hypertrophy, activation of pyruvate dehydrogenase kinases (PDK) followed by phosphorylate pyruvate dehydrogenase (PDH) inhibits the ability of CMs to catalyze pyruvate, therefore leading to a dependence on glycolysis for energy production [66]. Another adverse effect of the metabolic switch is the accumulation of reactive oxygen species (ROS) in CMs. HIF-1α activation leads to increased PDK activity, followed by mitochondrial complex II ROS generation [67,68,69]. Whereas previous studies revealed negative effects of ROS in the progression of HF [70,71], in animal models, reducing ROS during RV hypertrophy showed contradicting results [72,73]. While the response to ROS is similar between both ventricles during hypertrophy, the RV appears to be much more susceptible to changes in ROS homeostasis compared to the LV [74,75]. Increased intracellular ROS not only causes damage of proteins and DNA but also induces inflammation through the induction of pro-inflammatory chemokines, cytokines and transcription factors [76].

In PAH, the inflammatory response in the RV is marked by an increase in infiltrating CD45^+^/CD68^+^ cells [77] and pro-inflammatory cytokine signaling involving molecules such as tumor necrotic factor α (TNF-α) and interleuking-1 (IL-1) family members [78,79]. Whereas increased and sustained inflammation during RV hypertrophy appears to be beneficial at short term by leading to increased angiogenesis, it does affect CM functionality at long term, by decreasing contractility and increasing ROS production [80,81].

Despite many stimuli and regulatory mechanisms being identified and characterized, there is no cure yet available for PAH and RVH. Presently available therapies focus on symptomatic treatment and slowing down disease progression. The ultimate treatment option available is either lung transplantation, combined with RV support, or palliative support if long-term treatment options are no longer available or possible [82,83]. Looking at these outcomes, new areas in the biomedical field are being investigated in order to identify new potential therapeutic targets to reduce RV remodeling during PAH.

## 2. Epigenetic Regulation

### 2.1. Chromatin Modifications

The concept of epigenetic regulation was introduced in 1942 by Conrad Waddington, and initially described the process of the influence of genotypes on the phenotype during development [84]. However, later in 1996, Russo and colleagues, defined epigenetics more in line with our current definition: heritable changes in the genome that affect the phenotype without changing the DNA sequence [85]. Epigenetic inheritance functions in several distinct ways, including DNA methylation and histone modifications, but also through non-coding RNAs (ncRNAs), among others.

DNA methylation, which includes the process of new methylation, maintenance and removal of methyl groups, occurs at specific sites of the DNA, namely CpG or CG sites, where a cytosine is followed by a guanine base in the 5′ to 3′ direction. Conjugation with the methyl functional group is mainly catalyzed by two enzymes, DNA methyltransferases 3A and 3B (DNMT3A and DNMT3B, respectively), both consisting of two chromatin reading domains, PWWP and ATRX-DNMT3-DNMT3L (ADD), and the methyltransferase (MTase) domain [86]. Whilst cytosine enriched areas display higher methylation percentages, promoter areas of active genes are protected from methylation due to their enrichment in Histone 3 Lys4 trimethylation (H3K4me3) [87] as binding of the enzymatic ADD domain to the K4me3 tail prevents methylation of the promoter [88]. Binding of ADD to trimethylated H3K4 induces activation of the MTase domain, initiating the process of DNA methylation. While active promoters are free of methylated sites, the transcriptional region of active transcribed genes are enriched with methylated CpG sites [89]. During transcription by RNA polymerase II, another methyltransferase, SET domain containing 2 (SETD2), trimethylates H3K36, which is then able to bind PWWP and initiate methylation by DNMT3B [90]. In order to maintain methylation sites during DNA replication, two enzymes, DNA methyltransferase 1 (DNMT1) and ubiquitin-like, containing PHD and RING finger domains, 1 (UHRF1) need to work together [91]. UHRF1 binds to the methylated sites and then recruits DNMT1, which, unless bound to UHRF1 and similar to DNMT3, is autoinhibiting [92]. Demethylation occurs upon oxidation of the methylated cytosine to 5-carboxylcytosine (5caC), 5-hydroxymethylcytosine (5hmC) or 5-formylcytosine (5fC) by ten-eleven translocation (TET) methylcytosine dioxygenases [93]. These intermediate functional groups are then either removed by thymine DNA glycosylase (TDG) or passively diluted by DNA replication, leading to new unmethylated DNA strands [93,94,95]. While DNA methylation occurs at high rates, it has been observed that methylation is not present at transcription binding sites during binding [96] which, together with the ability of methylation to induce heterochromatin formation through DNMTs and lymphocyte-specific-helicase (LSH), gives rise to the function of DNA methylation as a transcriptional repressing process [97,98].

Histones are a group of proteins in the nuclei that organize and pack DNA. In 1964, the first experimental paper was released showing acetylation of histones and suggesting their role in RNA synthesis [99]. Acetylation of the lysine on the N-terminus leads to a removal of the lysine’s charge which, by weakening the bond between histones and DNA leads to easier accessibility of the DNA to proteins [100,101]. Eventually, additional studies showed lysine in histones to be targeted by two opposing enzymes: histone acetyltransferases (HATs) and histone deacetylase (HDACs) [102]. HATs can be divided into two main groups, type-A HATs and type-B HATs and within these two subgroups, the type-A HATs can be classified in three major families based on their conformation and amino-acid sequence: MOZ, Ybf2/Sas3, Sas2 and Tip60 (MYST), Gcn5-related N-acetyltransferases (GNAT) and p300/CREB-binding protein (p300/CBP) [103]. The type-A HATs are predominantly found in the nuclei but while the three families work through similar mechanisms starting with a deprotonation of the lysine’s amine followed by nucleophilic substitution facilitated by Acetyl CoA [104], they have different acetylation targets within the histones [105]. HDACs, in turn, deacetylate histones by a different mechanism that is not based on a cofactor but rather on zinc or NAD+ [106]. HDACs are either part of the histone deacetylation family or the Sirtuin protein family, and four classes can be distinguished based on the sequence similarity of the different enzymes [107]. Class I, II and IV are part of the same family and use zinc for their deacetylation mechanism, while class III HDACs’ use NAD+ [107]. During the reaction between acetylated histones and HDACs, zinc functions as a proton shuttle to stabilize the chemical binding and eventually the stable release acetate [108]. HDAC Class III enzymes act through a reduction/oxidation chemical reaction with the co-enzyme NAD+, and inducing transfer of acetyl groups from the lysine amino acid towards the ADP-ribose that is part the NAD+ co-enzyme [109]. The general consensus is that acetylation of histones, by allowing genes to be more accessible for transcription factor, increase gene expression [102].

Another histone modification is the phosphorylation of serines, threonines and tyrosines of in the N-terminus [110]. Momentarily, two functions were described for histone phosphorylation; those being DNA repair and transcription regulation [111]. Several histone phosphorylation sites, such as H3 serines 10 and 28 and H2B serine 32, have been linked to transcriptional regulation [112,113,114]. However, the role of phosphorylation on transcription regulation is not yet understood since some studies showed that phosphorylation of histones leads to recruitment of additional histone modification enzymes but others were not able to connect histone modification with the degree of phosphorylation [115,116,117].

**Figure 2:**
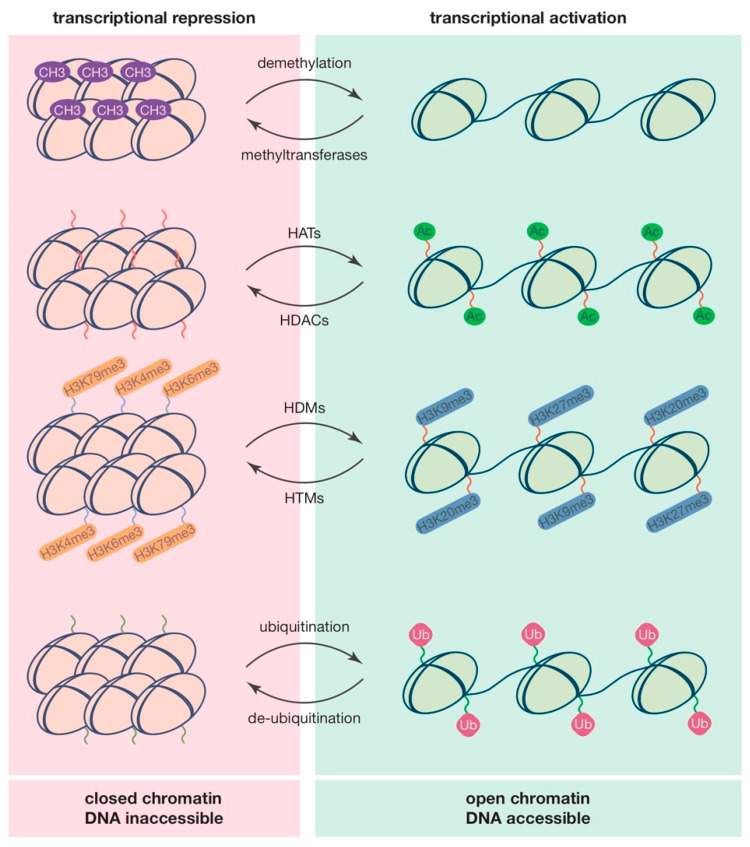
Epigenetic regulation and chromatin remodeling. Chromatin remodeling can be mediated by different DNA and/or histone modifications such as methylation, acetylation, and ubiquitination. Methylation of DNA is mediated mainly by DNMT3A and DNMT3B in cytosine rich locations. Methylated sites are seen as being transcriptionally inactive. In turn, histones can be either methylated, acetylated, or ubiquitinated. While methylation of DNA is a sign of transcription inhibition, methylation of histones by HMTs can either inhibit or enhance transcription depending on the amount of methylation occurring on the histones. Furthermore, acetylation of histone amino acids is a sign of transcriptional activation. HATs acetylate lysines while HDACs deacylated lysine, influencing transcription by increasing (acetylation) or decreasing (deacetylation) accessibility of genes. Ubiquitination of histones is another process that regulates gene expression. Ubiquitination occurs on both histone 2A lysine 199 and histone 3B lysine 200. The effect of ubiquitination on gene expression is depending on its location, histone 2A lysine 199 has a repressing effect while histone 2B 200 ubiquitination activates gene expression.

Histones, just like DNA, can also be methylated in order to regulate their expression. During methylation, the lysines and arginines of the histones can be conjugated without affecting the charge of the amino acids that are being methylated [118]. Unlike acetylation and phosphorylation, that only have one possible conjugation site, methylation can occur at multiple sites on the amino acids [119], in a process that is mediated by histone methyltransferases (HMTs) [120]. Methylation of histones can either enhance or inhibit transcription, depending on the number of methylated lysine/arginine sites per amino acid. For example, a monomethylated lysine leads to increased gene expression while a tri-methylated lysin represses gene expression [121,122]. A schematic overview of the regulation of chromatin remodeling by DNA methylation and major histone modifications is provided in Figure 2.

### 2.2. Chromatin Modifications in PAH

Chromatin modifications are able to regulate change gene expression and have gained much research interest initially due to their potential role in developmental biology [123,124,125]. Since gene expression is strongly associated to disease, is not a surprise that chromatin remodeling has also been studied in the context of PAH. HDAC class I activity was shown to increase during hypoxia-induced but in vivo HDAC inhibition resulted in decreased pulmonary remodeling during PAH [126]. Furthermore, in a rat model of PAH, inhibition of class I HDACs decreased proliferation and migration of PASMCs [127,128]. Other studies have involved HDACs in PAH-induced oxidative stress by showing reduced ROS and NAPDH oxidative (Nox) levels after HDAC inhibition [129]. Furthermore, histone methylation has recently also been implicated in PAH, more specifically in hypoxia-induced PAH, where megakaryocytic leukemia 1 (MKL1) is upregulated and leads to an increase in the recruitment of the H3K4-specific complex components, ASH2 and WDR5 [130,131]. Knocking out of the methylation complex in vivo resulted in improved vascular remodeling, compared to healthy controls [130].

DNA methylation has been described for the superoxide dismutase 2 (*SOD2*) gene, found to be downregulated in PASMCs of PAH patients [132]. A profiling study also revealed that vasculature-related genes are hypermethylated in PAH, showing that dysregulation of vascular maintenance is not only regulated through the three main signaling pathways discussed earlier, but also through epigenetic inheritance [133].

Since the leading cause of death during PAH is RV failure, therapies targeting PAH will also reduce pathological RV remodeling. There is, however, a distinct epigenetic roadmap for the different cardiac chambers [134] which accounts for contrasting results. There have also been studies observing that broad HDAC inhibitor trichostatin A (TSA) lead to worsening [135] while valproic acid (VPA) and MGCD0103 have positive effects on RV remodeling [126,128]. VPA is a known HDAC I inhibitor and in vivo shows reduced proliferation of adventitial fibroblasts while MGCD0103 inhibits proliferation of PASMCs. Both VPA and MGCD0103 function by inhibiting HDACs and their subsequent target FoxO3a. However, MGDC0103 is also able to reduce inflammation and apoptosis in the RV, leading to an increased functionality during disease [126].

While the mechanism of action behind HDAC inhibitors in PAH and RV remodeling has not yet been fully elucidated, unraveling it might help in achieving more specific therapies for the both PAH and RV failure (RVF).

### 2.3. Non-Coding RNAs

Until relatively recently, the central dogma of molecular biology assumed genomic DNA to be transcribed into messenger RNA (mRNA), which in turn would be translated into functional proteins [136]. Current knowledge reveals that only 2% of our genome is actually translated into proteins while the majority is composed of non-coding genetic information [137], non-coding transcripts (ncRNAs) that, for long, were described as transcriptional noise. However, multiple ncRNAs have been found to exert a regulatory effect in cellular pathways and therefore, being able to modulate disease onset and progression [138,139,140]. ncRNAs are classified based on their size and function, into miRNAs, circular RNAs (circRNAs) and long non-coding RNAs (lncRNAs). 

#### 2.3.1. MicroRNAs

miRNAs are short strands of RNA, between 20 and 24 nucleotides long, that regulate target mRNAs through sequence complementarity followed by either strand degradation or translational inhibition [141]. The first discovered miRNA, in *C. elegans*, in 1993, was described as an anti-sense RNA strand for the *lin-14* gene regulating the LIN-14 protein expression [142]. Nowadays we know that miRNAs are highly conserved among species, hinting at key regulatory functions [143]. Biogenesis of miRNAs starts with RNA polymerase II/III transcribing genomic DNA into a primary miRNA double-strand loop (pri-miRNA) that is then cleaved by the ribonuclease III enzyme DROSHA and RNA binding protein DiGeorge syndrome critical region 8 (DGCR8) complex into a pre-miRNA stem-loop, known as hairpin [144]. The pre-miRNA hairpin is then transported from the nucleus into the cytoplasm by two proteins, EXPORTIN5 and RanGTP. In the cytoplasm, cleavage by DICER, an RNase III endonuclease, into a mature miRNA duplex gives rise to two single strands, the 5p and 3p strand originating from the 5′ and 3′ ends of the hairpin [145]. One strand, the least stable, is thought to be degraded while the other strand is stabilized by the Argonaut complex. However, a recent study suggests that the least stable miRNA strand is shuttled out of the cells via extracellular vesicles and may play a role in cell-to-cell communication during disease progression [146]. The miRNA strand is then loaded into the RNA-induced silencing complex (RISC) where it will bind the 3′ untranslated region (UTR) of the target mRNA leading to either mRNA degradation or translational repression, depending on the complementarity between the two RNA strands [147]. The RISC includes the proteins Argonaut 2, DICER, TRBP, GW182 and PACT. While TRBP assists DICER in processing pre-miRNA, TRBP and PACT assist Argonaut 2 with binding to and stabilizing double stranded RNAs. Finally, GW182 helps Argonaut 2 in guiding the miRNA to ensure gene silencing [148].

#### 2.3.2. Circular RNAs

The first circRNA was discovered in 1976 by Sänger et al. when investigating viroids, uncoated circular RNA pathogens for plants [149]. However, it was not before 1990 that the first functional circRNA was described while investigating a tumor repressor gene [150]. Transcription of circRNAs is also initiated by RNA polymerase II to form a linear pre-mRNA strand. While multiple biogenesis routes are proposed, the most common method of circularization is when a 5′ splice donor and 3′ splice acceptor ends join. Both sites are usually flanked by large introns with repetitive sequences that that form a looping structure. Branchpoint nucleophilic attack during splicing allows for RNA circularization through 5′-3′ linkages, assisted by RNA binding proteins, such as Muscleblind or Quaking, or through complementary Alu sequences, short DNA sequences utilized by the restriction enzyme *Arthrobacter luteus* (Alu) [151,152]. The function of circRNAs can differ depending on their sequence and subcellular localization. When in the nucleus, circRNAs can either enhance or inhibit transcription of parental genes [153,154]. circRNAs are also able to sponge miRNAs in the cytoplasm to prevent target mRNA degradation as it was shown for the conserved *ciRS-7*, with more than seventy miR-7 binding sites [155]. The study showed that when overexpressing *ciRS-7* in vitro, addition of miR-7 resulted in lower silencing efficiency of known targets. Similarly, in zebrafish, an increase in *ciRS-7* leads to an impairment in the midbrain, similar to what was observed when knocking out miR-7 [156]. Another circRNA, sex-determining region Y (*Sry*), was shown to function as a sponge for miR-138 in vitro with similar results as *ciRS-7* when increased in vitro [155]. 

Furthermore, circRNAs are able to interact with RNA binding proteins and create a scaffold for enhanced RBP-RNA interactions, Argonaute being one example for this interaction [153,155]. A study performed on *ciRS-7* showed that it does not only have miR-7 binding sites, but also contains binding sites used by Argonaute 2, assisting with the gene silencing induced by miR-7 [156]. While circRNAs are classified as non-coding, recent studies show that they are able to produce proteins and peptides. Such examples are *circZNF609* which translates into a protein and polypeptides with a currently unknown function, and *circ*β*-catenin* which translates into a novel β-catenin isoform that stabilizes the full-length linear β-catenin by inhibiting GSK3β-mediated degradation [157,158,159].

#### 2.3.3. Long Non-Coding RNAs

lncRNAs, named according to their length of a minimum of 200 nucleotides, were first described in 1990 with *H19* being the first lncRNA to be characterized, followed by X-inactive specific transcript (*XIST*) in 1992 [160,161,162]. lncRNAs are associated with a plethora of functions related to regulation of transcription and/or translation, cell cycle homeostasis, cell differentiation and cell senescence [163,164,165,166,167]. In the nucleus, lncRNAs regulate gene expression through chromatin remodeling by providing protein scaffolds to guide enzymes such as acetylases, histone and DNA methyltransferases, towards specific locations in the genomic DNA [168,169]. They can also enhance a specific gene promoter by recruiting RNA binding proteins or act as co-factors for specific transcription factors [170,171]. 

**Figure 3:**
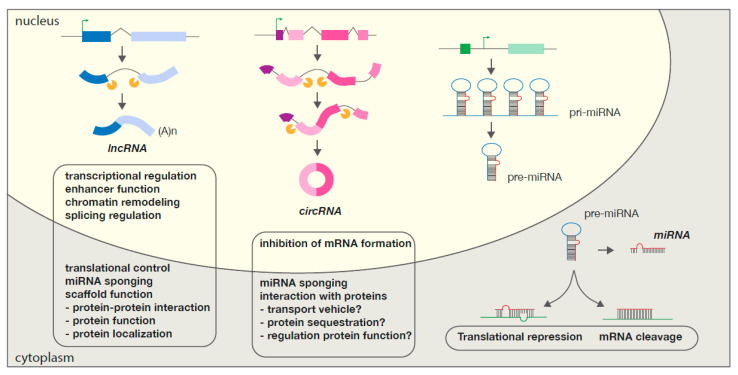
Different ncRNA species: their transcription from the eukaryotic genome and respective functions. Exons in protein-coding genes can be transcribed into lncRNAs (blue) and circRNAs (purple), normally after intron exclusion by the spliceosome (orange). miRNAs (green), in turn, can derive from intronic sequences or intergenic regions. LncRNAs are a large class of transcribed RNA molecules with a length of more than 200 nucleotides that are mostly located exclusively in the nucleus. They are often transcribed as whole or partial antisense transcripts to coding genes and can structurally resemble mRNAs. They exert their function by binding DNA, RNA, or proteins, to regulate gene expression at multiple levels via base pairing or secondary structure formation, respectively. LncRNAs, by acting as either signals, decoys, guides, and/or scaffolds, can recruit epigenetic factors to change patterns of chromatin organization, activate or repress the gene transcription by interacting with specific regulatory regions or transcription factors and manipulate mRNA function by regulating alternative splicing. At a posttranscriptional level lncRNAs can act as competing endogenous RNA, by base pairing with miRNAs and interfering with their inhibitory effects, but they can also affect mRNA translation and can modify mRNA and proteins, playing key roles in protein-protein interactions, protein activity and localization. While many different circRNAs can be generated from a single genomic locus, all of them are generated through backsplicing, a non-canonical splicing process in which a downstream splice donor is joined to an upstream splice acceptor. The roles of circRNAs are poorly described and remain therefore limited to inhibition of a linear, functional mRNA formation and microRNA sponging. They have been shown to also interact with proteins and although the exact function of such interactions remains unclear, they are speculated to be involved in either protein transport, protein sequestration and/or protein regulation. miRNAs are transcribed from intronic sequences and form a double stranded loop primary miRNA (pri-miRNA). Further processing generates precursor miRNA molecules (pre-miRNA) which once in the cytoplasm will produce mature miRNA molecules. Association of the miRNA with large protein complexes (RISC) allows for contact with target mRNA sequences. miRNAs will inhibit mRNA translation when a near-perfect pairing occurs or induce full mRNA cleavage with a perfect pairing.

While regulation of gene transcription is a potent mechanism to control gene expression, post-transcriptional modifications should not be overlooked. lncRNAs function through multiple ways to interact and affect gene expression post-transcriptionally, for example by sponging local miRNAs to prevent mRNA degradation, or by hosting specific miRNAs to increase their levels in the cell [172,173]. The lncRNA *Xist*, originating from the X-inactivation center (XIC), is able to inactivate one X-chromosome in female mammalian embryos [174]. *Xist* coats the chromosome in cis by activating polycomb-groups (PcGs), a group of DNA methyltransferases, that will induce H3K27 trimethylation by interacting with the 5′ end of *Xist*, containing an A-repeat. This eventually leads to formation of heterochromatin and subsequent silencing of the chromosome. *Xist* itself is regulated by *Tsix*, a lncRNA overlapping with Xist but translated antisense, in cis [175]. An overview of the classes of ncRNA, their biogenesis and cellular function is provided in Figure 3.

### 2.4. Non-Coding RNAs Involved in PAH

ncRNAs have been associated with a prominent role in disease progression [176,177,178,179] and multiple studies revealed differential miRNA expression during the development of PAH. Multiple miRNAs were found to be dysregulated among different vascular cell types, allowing for clustering per cell class [180]. In VSMCs, the miR-17~92 cluster, miR-21, miR-145 and miR-210 are upregulated while miR-124 and miR-204 are downregulated. In ECs, the miR-17~92 cluster and miR-27a are upregulated but miR-21, miR-424 and miR-503 are downregulated. miR-340-5p was shown to be downregulated after acute pulmonary embolism and its overexpression in vivo prevented the development of PAH in a rat model of embolism through direct targeting of both interleukin-6 (IL-6) and interleukin-1β (IL-1β) and subsequent activation of nuclear factor κβ (NF-κβ) in PASCMs [181]. miR-138-5p is upregulated in PASMCs from PAH patients as well from rats subjected to MCT-induced PAH and its inhibition restores *KCNK3* and Solute carrier family 45-member 3 (*SLC45A3*) expression and ameliorates PAH and RV function [182]. While *SLC45A3′s* function in PAH is not yet elucidated, *KCNK3* inhibitions leads to increased proliferation, inflammation and subsequent vasoconstriction of the pulmonary artery [183]. In PAH patients, PASMC function is also tightly controlled by upregulated miR-18a-5p, through targeting of Notch2. While in PAH patients, miR-18a-5p influences PASMC proliferation and migration [184]. In MCT-treated rat PASMCs metabolism is affected by miR-125a-5p, through targeting of hexokinase 2 (HK2) and subsequent decrease in glycolysis [185].

However, not only SMCs are affected during PAH. Pulmonary fibroblasts are also affected during PAH but show only one miRNA to be downregulated, miR-124 [180]. With a plethora of miRNAs available, multiple therapeutic approaches are possible and some have already been tested. Studies where miR-145 was silenced, showed promising results by reducing pulmonary and subsequent RV remodeling in a hypoxia model [186]. Furthermore, circulating miRNAs have been investigated as biomarkers for PAH, and miRNAs such as miR-21-5p, miR-22-3p and miR-451a have been established as potential diagnostic tools to assess the development and/or progression of PH [187]. 

To date, only one circRNA, *hsa_circ_0016070*, has been involved in the pathophysiology of PAH. PASMCs transfected with *hsa_circ_0016070* showed an increase in proliferation by inhibition of cell cycle arrest. It should be noted that in vivo validation of these results are lacking [188]. However, recently, increased levels of *CircATP2B4* were detected in serum of PAH patients and associated with SMCs proliferation and migration. Bioinformatics and in vitro testing showed that *CircATP2B4* is able to sponge miR-223, leading to an increase of the ATR protein, increased PASMC proliferation/migration and decreased apoptosis [189]. Further studies are expected to identify many other circRNAs involved in PAH and pulmonary arterial remodeling.

**Figure 4:**
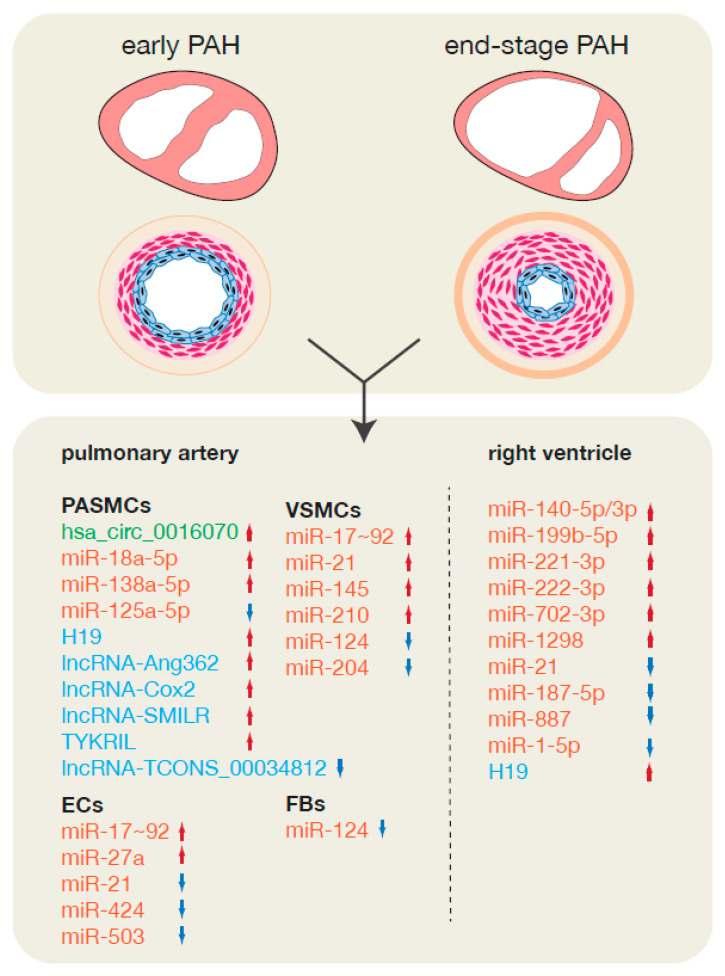
ncRNAs involved in PAH-induced pulmonary artery and RV remodeling. During disease progression, multiple ncRNAs are dysregulated in the different vascular and cardiac cell types, and compared to healthy tissues. miRNAs are depicted in red, lncRNAs in blue and circRNAs in green. Red and blue arrows represent gene up- or downregulation, respectively, in either the pulmonary artery or the RV.

lncRNAs have also been investigated as possible regulatory players during PAH-induced remodeling and multiple profiling studies have been performed to elucidate the role of lncRNAs in PAH [190,191,192,193,194]. In MCT rat models, the developed inflammatory response leads to an increase of *H19* in the lungs and subsequent vascular remodeling. Increased *H19* is able to sponge the *let-7b* leading to an increase of its downstream target, Angiotensin II receptor type 1 (*AT_1_R*), ensuing an increase in PASMC proliferation. This was confirmed by in vivo studies where a *H19* null mice was subjected to MCT-induced PAH and reduced arterial remodeling was observed [190]. More recently, LncRNA-*TCONS_00034812* was detected in the pulmonary artery of rats exposed to chronic hypoxia and by being downregulated in PASMCs it increases their proliferative capacity while reducing apoptosis, through decreased phosphorylation of p-ERK, p-JNK and p-p38 [191]. Another lncRNA, *lnc-Ang362*, was discovered to be upregulated together with miR-221 and miR-222 in the lung vasculature from patients suffering from PAH. Further in vitro studies showed that *lnc-Ang362* regulates both miR-221 and miR-222 expression with its knockdown resulting in downregulation of miR-221 and miR-222 in PASMCs. Upregulation of *lnc-Ang362* leads to increased PASMCs proliferation and migration which was reduced when miR-221 or miR-222 were inhibited through *lnc-Ang362* overexpression [193]. Another lncRNA, *lincRNA-Cox2*, has been described as a regulator of inflammation, involved in atherosclerosis, and also found to be involved in PASCM migration and proliferation. *LincRNA-Cox2* sponges miR-let-7a and leads to an increase in STAT3 [195]. In patient peripheral blood samples, *lincRNA-Cox2* is upregulated, which is also observed in vitro in hypoxic PASMCs, and its silencing leads to decreased cell proliferation and migration. Furthermore, PASMCs derived from iPAH patients showed an increase in *TYKRIL*, a lncRNA playing a role in pericyte survival and vascular remodeling through regulation of Platelet-derived growth factor receptor beta (PDGFRβ) [196]. Another lncRNA that is upregulated in PAH patients and in vivo models and shown to be involved in vascular remodeling is *lncRNA-SMILR*. By sponging miR-141, *lncRNA-SMILR* leads to increased expression of RhoA/ROCK [197] and alterations in the proliferative and migratory capacity of PASMCs. An overview of the different ncRNAs that were associated with PAH is represented in Figure 4.

While the studies by Wang et al. and Lei et al. were performed using patient material, most studies so far have not been validated in patients. While it remains to be proven that lncRNA expression patterns and function is conserved towards humans by being a new and upcoming field, there is a plethora of ncRNAs detected differentially expressed in PAH that might lead to a better understanding of this deadly disease, as well as to the development of more efficient therapeutic strategies.

### 2.5. Non-Coding RNAs in Right Ventricular Remodeling

Looking more specifically at RV hypertrophy, not much is known about specific ncRNAs involved in RV remodeling compared to the information available for LV remodeling. Most studies remain descriptive by merely showing differences either between LV and RV or healthy and disease RV [198,199,200]. Some miRNAs involved in RV hypertrophy such as miR-21, miR-140-5p and -3p, miR-187-5p, miR-199b-5p, miR-221-3p, miR-222-3p, miR-702-3p, miR-887 and miR-1298 appear to have ventricle specific responses to pressure overload and remodeling [201,202,203]. For example, while in the LV miR-21 exerts a protective role during ventricle remodeling, increased miR-21 levels during RV remodeling correlate with severity of RV hypertrophy [201,202]. In turn, miR-199b is upregulated in both ventricles under pressure overload, but its target genes are differentially affected with DYRKA1 being downregulated in the LV while not being altered in the RV [204,205]. Furthermore, miR-1-5p has been described to be decreased in RV hypertrophy with its target, transforming growth factor beta receptor I (*TGFβR1*) being upregulated in a MCT rat model. However, in vivo studies including modulation of either miR-1-5p or *TGFβR1* in MCT rats to validate the importance of the miR-1-5p during RVH are still lacking [206]. Figure 4 depicts the specific ncRNAs that were found to play a role in RV remodeling.

While therapeutic intervention targeting RV-specific miRNAs as not yet been achieved, researchers have been focusing on pulmonary specific miRNAs, reducing the triggers for RV remodeling [207]. In the past decade, interest in RV-specific lncRNAs has increased as reflected by the several profiling studies performed on patient material and/or animal models [198,207,208].

Although one study has compared the role of *H19*, in both LV and RV remodeling induced by pressure overload and shown that of *H19* in the LV protects against hypertrophic remodeling while in the RV *H19* exacerbates the hypertrophic response [209,210], most studies remains descriptive. Without focusing on the mechanisms, the contribution of such lncRNAs towards the development and progression of the disease will remain unclear. 

## 3. Future Perspectives

Over the last two decades many improvements have been made towards treatment and symptom management of PAH patients but nevertheless, no cure has been found yet. Therefore, by exploring new fields such as epigenetic regulation, may help in better understanding the disease mechanisms as well as in developing new diagnostic and therapeutic strategies. However, many questions still remain: is ncRNA function conserved in humans and does it change in a similar way towards disease progression? If so, can we target specific cell types or tissue? Among many other questions that hopefully we will be able to answer in the upcoming decade.

Another possibility may be that we do not focus on PAH itself but rather on its clinical outcome, RVF. We currently know that while both ventricles share the same cellular composition, intracellular signaling pathways are different within these two cardiac chambers, but it is not yet understood whether this is a result of the cell’s regulatory machinery or imprinting during embryonic development [211]. Finally, it is safe to say that a very exciting and exploratory field remains to be unveiled to further elucidate the mechanisms behind cardiac hypertrophy in general and the RV remodeling in response to PAH specifically.

## Abbreviations

5caC	5-Carboxylcytosine
5fC	5-Formylcytosine
5hmC	5-Carboxylmethylcytosine
ADD	ATRX-DNMT3-DNMT3L
Alu	*Arthrobacter luteus*
AT1R	Angiotensin II Receptor Type 1
ATP	Adenosine triphosphate
ATP13A3	Probable cation-transporting ATPase 13A3
ACVRL1	Serine/threonine-protein kinase receptor R3
AQP1	Aquaporin 1
BMPR2	Bone morphogenetic protein receptor type II
cAMP	Cyclic adenosine monophosphate
CAV1	Caveolin
cGMP	Cyclic 3′,5′ guanosine monophosphate
circRNA	Circular RNA
CM	Cardiomyocyte
DGCR8	DiGeorge Syndrome Critical Region 8
DNMT1	DNA methyltransferases 1
DNMT3A	DNA methyltransferases 3A
DNMT3B	DNA methyltransferases 3B
ECs	Endothelial cells
EIF2AK4	Eukaryotic translation initiation factor 2-alpha kinase 4
eNOS	Endothelial nitric oxide synthase
EPAC1	Exchange factor directly activated by cAMP
ERK1/2	Extracellular signal-regulated kinases 1/2
ET-1	Endothelin-1
FBs	Fibroblasts
FFA	Free fatty acid
GDF2	Growth differentiation factor 2
GLUT1	Glucose transporter 1
GLUT4	Glucose transporter 4
GNAT	Gcn5-related N-acetyltransferases
GPCRs	G-coupled protein receptor
H3K4me3	Histone 3 Lys4 trimethylation
HAT	Histone acetyltransferase
HDAC	Histone deacetylase
HIF-1α	Hypoxia-inducible factor 1-alpha
HK2	Hexokinase-II
HMTs	Histone methyltransferase
hPAH	Hereditary pulmonary artery hypertension
IL-1	Interleukin-1
IL-1β	Interleuking-1β
IL-6	Interleuking-6
IP	Prostaglandin receptors
JNK	c-Jun N-terminal kinase
KCNK3	Potassium two pore domain channel subfamily K member 3
lncRNA	lncRNA
LSH	Lymphocyte-specific-helicase
LV	Left ventricle
MCT	Monocrotaline
miRNA	MicroRNA
MKL1	Megakaryocytic leukemia 1
mPAP	Mean pulmonary arterial pressure
mRNA	messenger RNA
MTase	Methyltransferase
MYST	Moz, Ybf2/Sas3, Sas2 and Tip60
ncRNA	Non-coding RNA
NF-κβ	Nuclear Factor κ β
NO	Nitric oxide
Nox	NAPDH oxidative
p300/CRBP	p300/CREB-binding protein
PAB	Pulmonary artery banding
PAH	Pulmonary Artery Hypertension
PASMCs	Pulmonary artery smooth muscle cells
PcGs	Polycomb-groups
PCWP	Pulmonary capillary wedge pressure
PDE5	Phosphodiesterase type 5
PDGFRβ	Platelet-Derived Growth Factor Receptor beta
PDH	Pyruvate dehydrogenase
PGI2	Prostacyclin
PH	Pulmonary hypertension
PKA	Protein Kinase A
PKC	Protein Kinase C
PKD	Pyruvate dehydrogenase kinases
PLC	Phospholipase C
pri-miRNA	Primary miRNA double-strand loop
PVR	Pulmonary vascular resistance
RHF	Right heart failure
RhoA/ROCK	RhoA/Rho-Kinase
RISC	RNA Induced Silencing Complex
ROS	Reactive oxygen species
RV	Right ventricle
RVF	Right ventricle failure
RVH	Right ventricle hypertrophy
SETD2	SET Domain containing 2
sGC	Soluble guanylate
SLC45A3	Solute Carrier Family 45 Member 3
SMAD1	Mothers against decapentaplegic homolog 1
SMAD4	Mothers against decapentaplegic homolog 4
SMAD9	Mothers against decapentaplegic homolog 9
SMCs	Smooth muscle cells
SOD2	Superoxide dismutase 2
SOX17	SRY-box 17
Sry	Sex-determining region Y
TBX4	T-box transcription factor 4
TDG	Thymine DNA glycosylase
TET	Ten-eleven translocation
TGF-β	Transforming growth factor beta
TGF-βR1	Transforming growth factor beta receptor I
TNF-α	Tumor necrotic factor alpha
TSA	Trichostatin A
UHRF1	Ubiquitin-line, containing PHD and RING finger domains, 1
UTR	Untranslated region
VPA	Valproic acid
XIC	X-inactivation center
XIST	X-inactive specific transcript
